# Highly prevalent bartonellae and other vector-borne pathogens in small mammal species from the Czech Republic and Germany

**DOI:** 10.1186/s13071-019-3576-7

**Published:** 2019-07-03

**Authors:** Anna Obiegala, Kathrin Jeske, Marie Augustin, Nina Król, Stefan Fischer, Katja Mertens-Scholz, Christian Imholt, Josef Suchomel, Marta Heroldova, Herbert Tomaso, Rainer G. Ulrich, Martin Pfeffer

**Affiliations:** 1 Institute of Animal Hygiene and Veterinary Public Health, University of Leipzig, Leipzig, Germany; 2 Institute of Novel and Emerging Infectious Diseases, Friedrich-Loeffler-Institut, Federal Research Institute for Animal Health, Greifswald-Insel Riems, Germany; 3 Institute of Bacterial Infections and Zoonoses (IBIZ), Friedrich-Loeffler-Institut, Federal Research Institute for Animal Health, Jena, Germany; 4 Julius Kühn-Institute, Federal Research Institute for Cultivated Plants, Institute for Plant Protection in Horticulture and Forests, Vertebrate Research, Münster, Germany; 5 Faculty of AgriSciences, Department of Zoology, Fisheries, Hydrobiology and Apiculture, Mendel University in Brno, Zemědělská 1, 613 00 Brno, Czech Republic; 6 Department of Forest Ecology, Mendel University in Brno, Zemědělská 3, 613 00 Brno, Czech Republic

**Keywords:** *Anaplasma*, *Apodemus*, *Babesia*, *Bartonella*, “*Candidatus* Neoehrlichia mikurensis”, *Coxiella burnetii*, *Microtus*, *Myodes*, Rodent, Shrew

## Abstract

**Background:**

Rodents are important reservoirs for zoonotic vector-borne agents. Thus, the distribution of rodents and their vicinity to humans and companion animals may have an important impact on human and animal health. However, the reservoir potential of some rodent genera, e.g. *Microtus*, has not yet been precisely examined concerning tick-borne pathogens in Central Europe. Therefore, we examined small mammals from Germany and the Czech Republic for the following vector-borne pathogens: *Babesia* spp., *Bartonella* spp., *Anaplasma phagocytophilum*, “*Candidatus* Neoehrlichia mikurensis” (CNM) and *Coxiella burnetii*. Spleen DNA from 321 small mammals belonging to four genera, *Myodes* (*n* = 78), *Apodemus* (*n* = 56), *Microtus* (*n* = 149), *Sorex* (*n* = 38), collected during 2014 in Germany and the Czech Republic were available for this study. DNA samples were examined for the presence of *Babesia* and *Bartonella* DNA by conventional PCR targeting the *18S* rRNA gene and the 16S–23S rRNA intergenic spacer region, respectively. For the detection of CNM, *A. phagocytophilum* and *C. burnetii* real-time PCR assays were performed.

**Results:**

*Bartonella* spp. DNA was detected in 216 specimens (67.3%) with 102/174 (58.6%) positive in Germany and 114/147 (77.6%) in the Czech Republic. The prevalence in each genus was 44.9% for *Myodes*, 63.2% for *Sorex*, 77.2% for *Microtus* and 75% for *Apodemus.* Four *Bartonella* species, i.e. *Bartonella* sp. N40, *B. grahamii*, *B. taylorii* and *B. doshiae*, as well as uncultured bartonellae, were detected. The *Bartonella* species diversity was higher in rodents than in shrews. In total, 27/321 (8.4%) small mammals were positive for CNM and 3/321 (0.9%) for *A. phagocytophilum* (*S. coronatus* and *M. glareolus*). All samples were negative for *Babesia* spp. and *Coxiella* spp.

**Conclusions:**

While the detected high prevalence for *Bartonella* in *Apodemus* and *Myodes* spp. is confirmatory with previous findings, the prevalence in *Microtus* spp. was unexpectedly high. This indicates that individuals belonging to this genus may be regarded as potential reservoirs. Interestingly, only *Sorex* spp. and *M. glareolus* were positive for *A. phagocytophilum* in the present study, suggesting a possible importance of the latter for the maintenance of certain *A. phagocytophilum* strains in nature.

**Electronic supplementary material:**

The online version of this article (10.1186/s13071-019-3576-7) contains supplementary material, which is available to authorized users.

## Background

Rodents and other small mammals are important reservoir hosts for a range of pathogenic and non-pathogenic viral, bacterial and parasitic agents [1]. They are of importance for the development of subadult tick stages and contribute in the natural life-cycle of several tick-borne bacterial and parasitic pathogens [2]. Thus, the distribution of rodents and their close contact to humans and companion animals may have impact on the health status of the latter. Bartonellae are known to infect endothelial cells and erythrocytes of mammals and humans [3]. The most common causative agent for bartonellosis in humans, *Bartonella henselae*, is mainly harboured by wild and domestic cats [4]. However, rodents are known to be the main reservoirs for the majority of over 22 species and subspecies of the already described bartonellae [5]. Nevertheless, although zoonotic bartonellae are confirmed to be harboured by rodents, the pathogenic potential is still unknown for most of them [5]. In Europe, *Bartonella* spp. were thus far reported in different vole and mice species from Austria, Finland, Germany and Poland [6–9].

“*Candidatus* Neoehrlichia mikurensis” (CNM) as well as *Anaplasma phagocytophilum* are both tick-borne alpha-proteobacteria [10]. While *A. phagocytophilum* has zoonotic potential and is responsible for a broad spectrum of symptoms in humans as well as in companion animals, CNM seems to be a health risk mainly in immunosuppressed humans as well as in dogs, causing mostly mild symptoms [11, 12]. In Europe, mainly rodents belonging to the genera *Myodes* and *Apodemus* are regarded as reservoirs for CNM. Specimens belonging to the genus *Microtus* have tested positive, but thus far they have only been examined in small sample sizes (*n* < 24) [13–15]. In central Europe, most rodent species are regarded as accidental hosts for *A. phagocytophilum* [16]. Nevertheless, it is yet not known whether rodents belonging to the genus *Microtus* are potential reservoirs [17].

*Coxiella burnetii*, the causative agent of Q fever, is a coccoid, obligate intracellular pathogen belonging to the order Legionellales and the family *Coxiellaceae*. Ticks may transfer *C. burnetii* to humans and mammals. The causative agent of Q fever may persist in endemic areas in reservoir hosts such as small mammals [18].

Several small mammal species of the genera *Myodes*, *Apodemus* and *Microtus* are supposed to be reservoirs for the tick-borne protozoan *Babesia microti* (order Piroplasmida, family Babesiidae) in Europe [17, 19]. Nonetheless, human babesiosis caused by *B. microti*, displaying various symptoms, has been rarely reported in Europe [20]. As data on the aforementioned vector-borne pathogens in small mammals from central Europe are scarce, the aims of this study were: (i) to evaluate the presence of *Bartonella* spp., CNM, *A. phagocytophilum*, *Babesia* spp. and *Coxiella burnetii* in small mammals captured in Germany and the Czech Republic; and (ii) to compare and analyse differences in the prevalence of these pathogens between small mammal species in connection with weight and age in order to evaluate the respective potential reservoir roles.

## Methods

### Collection of small mammal samples

A total of 321 small mammals belonging to nine different species [*Apodemus agrarius* (*n* = 2); *A. flavicollis* (*n* = 48); *A. sylvaticus* (*n* = 6); *Microtus agrestis* (*n* = 1); *M. arvalis* (*n* = 148); *Myodes glareolus* (*n* = 78); *Sorex araneus* (*n* = 30); *S. coronatus* (*n* = 7); and *S. minutus* (*n* = 1)] were collected for a previous study [21] (Table 1). Out of 148 *M. arvalis*, 147 individuals were collected according to standard protocols during late fall 2014 at three grassland grids close to Brno, the second largest city of the Czech Republic, located in the south-east. A further 174 individuals of different species were collected during spring, summer and fall in 2014 at grassland and forest grids at three sites in Germany [21]. The age of *Microtus* spp. was categorized in three classes according to the animals’ body weight: (1) < 14 g (less than 1.5 months-old); (2) 14–19 g (1.5 to 2.5 months old); and (3) > 19 g (2.5 months and older). Accordingly, the age categories in relation to the body weight for *Apodemus* spp. were classified as follows: (1) < 20 g (less than 3.5 months-old); (2) 20–30 g (3.5 to 7 months-old); and (3) > 30 g (7 months and older). For *M. glareolus* they were: (1) < 15 g (less than 1.5 months-old); (2) 15–19.5 g (1.5 to 2.5 months-old); and (3) > 19.5 g (2.5 months and older) [22]. Individuals belonging to body weight classes 1 and 2 were considered as sub-adults and individuals belonging to class 3 as adults. For *S. araneus* and *S. coronatus*, 2 categories were determined: (1) weight class < 8 g as sub-adult, and (2) weight class > 8 g as adult [23].

**Table 1:** *Bartonella* spp., *Anaplasma phagocytophilum* and “*Candidatus* Neoehrlichia mikurensis” in small mammals from Germany and the Czech Republic

Small mammal family/species	No. of analysed small mammals			No. of small mammals positive (%; 95% CI^a^) for		
	Total	Females	Males	*Bartonella* spp.	*A. phagocytophilum*	CNM^b^
Muridae^c^	56	26	30	42 (75; 62.2–84.6)	0 (0)	8 (14.3; 7.2–26.0)
* Apodemus agrarius*	2	1	1	2 (100; 29.0–100)	0 (0)	0 (0)
* Apodemus flavicollis*	48	23	25	36 (75; 61.1–85.2)	0 (0)	6 (12.5; 5.5–25.1)
* Apodemus sylvaticus*	6	2	4	4 (66.7; 29.6–90.8)	0 (0)	2 (33.3; 9.3–70.4)
Cricetidae	227^d^	118^d^	108^d^	150 (66.1; 59.7–71.9)	1 (0.4; 0–2.7)	19 (8.4; 5.4–12.8)
* Microtus agrestis* ^c^	1	1	0	0 (0)	0 (0)	0 (0)
* Microtus arvalis* ^e^	148^d^	77^d^	70^d^	115 (77.7; 70.3–83.7)	0 (0)	7 (4.7; 2.1–9.6)
* Myodes glareolus* ^c^	78	40	38	35 (44.9; 34.3–55.9)	1 (1.3; 0–7.6)	13 (16.7; 9.9–26.6)
Soricidae^c^	38	17	21	24 (63.2; 47.3–76.7)	2 (5.3; 0.5–18.2)	0 (0)
* Sorex araneus*	30	12	18	19 (63.3; 45.5–78.2)	0 (0)	0 (0)
* Sorex coronatus*	7	4	3	5 (71.4; 35.2–92.4)	2 (28.6; 7.6–64.8)	0 (0)
* Sorex minutus*	1	1	0	0 (0)	0 (0)	0 (0)
Total	321	161	159	216 (67.3; 62.0–72.2)	3 (0.9; 0.2–2.8)	27 (8.4; 5.8–12.0)

^a^95% CI, 95% confidence interval

^b^“*Candidatus* Neoehrlichia mikurensis”

^c^All derived from Germany

^d^Sex could not be determined for one individual

^e^147 individual derived from the Czech Republic, 1 individual from Germany

### Preparation of spleen DNA samples for molecular biological examination

Spleen-derived DNA samples of each individual were isolated separately as described previously [21] and were determined in terms of quantity and quality by the use of a spectrophotometer (Nano Drop ND-1000; PeqLab, Erlangen, Germany). As erythrocytes are the target cells of invasion and replication for *Bartonella* spp., spleen was chosen as the target tissue due to its important role with regard to removing old erythrocytes, and may thereby hold a reserve of erythrocytes that are highly infected by non-replicating bartonellae [24]. DNA samples with a concentration > 40 ng/µl were diluted with water (bioscience grade, nuclease-free) using different dilution steps in order to receive approximately equal DNA amounts between 20 and 40 ng/µl for further usage in PCR.

### Detection of *Bartonella* spp., *Babesia* spp., “*Candidatus* Neoehrlichia mikurensis”, *Coxiella burnetii* and *Anaplasma phagocytophilum via* real-time and conventional PCR

For the detection of *Bartonella* spp., a conventional PCR targeting a fragment of the 16S–23S rRNA ITS region [453–780 base pairs (bp)] was performed as described [25, 26]. A conventional PCR targeting the *18S* rRNA gene (411–452 bp) was performed for the detection of *Babesia* spp. [27] with slight modifications [25]. Obtained amplicons for both pathogens were separated by electrophoresis in 2% agarose gels, and visualized with HDGreen Plus DNA Stain (Intas Science Imaging Instruments GmbH, Göttingen, Germany) under UV-light. PCR products were purified using the NucleoSpin® Gel and PCR clean-up kit (Macherey-Nagel GmbH & Co. KG, Düren, Germany) according to the manufacturer’s instructions, and sequenced commercially (Sanger method) with forward and reverse primers (Interdisziplinäres Zentrum für Klinische Forschung, Leipzig, Germany). Sequences were analysed with BioNumerics v.7.6 (Applied Maths NV, Austin, TX, USA) and aligned to sequences obtained in GenBank using BLASTn (National Center for Biotechnology Information, Bethesda, MD, USA). A speciation cut-off was set at 98%. A selection of sequences (n = 50) was uploaded to GenBank under following accession numbers: MN056364-MN056413.

To detect CNM, a real-time PCR was performed targeting a 99-bp-sized fragment of the *groEL* gene [14] with modifications as described [16]. For detection of *A. phagocytophilum* a real-time PCR was performed targeting the *msp2* gene (77 bp) [28, 29]. Presence of *C. burnetii* was evaluated *via* real-time PCR targeting the single copy *icd* gene as described previously [30]. Briefly, DNA samples were tested and compared to *icd* plasmid standards ranging from 10 to 10^6^ copies/µl. All samples with > 10 copies/µl (detection limit) were considered positive. Details on primers are given in Additional file 1: Table S1.

### Statistical analysis

Confidence intervals (95% CI) for prevalences of the various pathogens were determined by the Clopper and Pearson method using Graph Pad Prism Software v. 4.0. (Graph Pad Software Inc., San Diego, CA, USA).

Host specificity was modelled using a generalized linear model (GLM using package *lme4*) with binomial error distribution where individual infection probability depended on the respective species. To estimate species-specific infection probabilities, estimated marginal means were obtained from the *emmeans*-package. After back transformation from logit scale-based on the reference GLM, the resulting infection probabilities were used to visualize host specificity. Only species with more than 10 trapped individuals were incorporated into the analysis. Similarly, binomial GLM’s were used to identify if certain demographic groups were particularly prone to infection. Here, sex (binary) and weight (continuous; used as surrogate for age) were used to predict individual infection status. Backwards parameter selection was performed using the *drop1* function. All analyses were performed using R [31].

## Results

### PCR results and sequence analysis for *Bartonella* spp. in small mammals

**Fig. 1:**
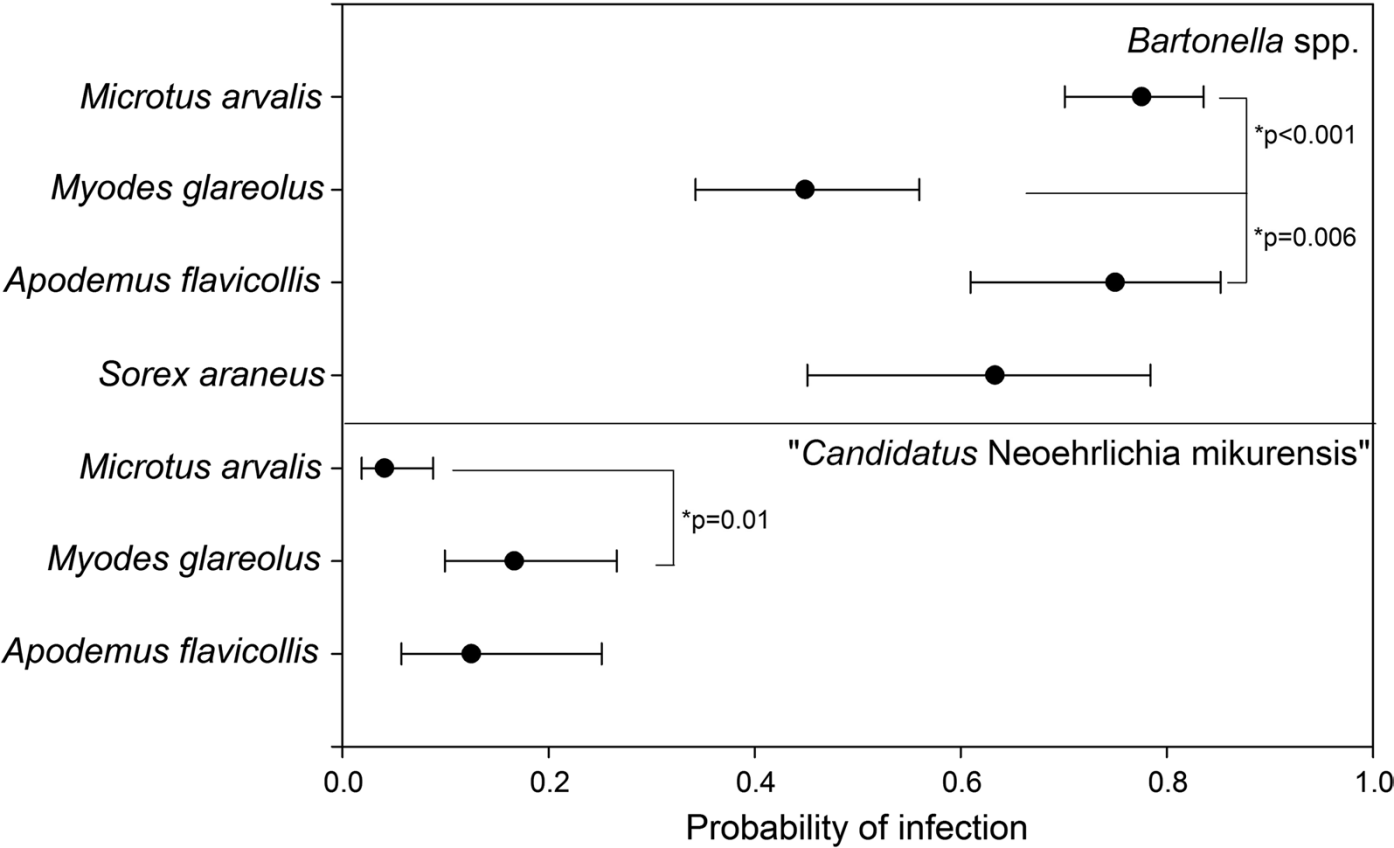
Results of generalized linear models for species specific infection probabilities for *Bartonella* spp. and CNM infections. *P*-values were obtained from *post-hoc* analysis (Tukey’s test)

In total, 216 out of 321 individuals (67.3%; 95% CI: 62.0–72.2%) were positive for *Bartonella* spp. DNA, with 102/174 (58.62%; 95% CI: 51.19–65.68%) from Germany and 114/147 individuals (77.6%; 95% CI: 69.94–84.02%) from the Czech Republic (Table 1). The prevalence also differed between host genera (*χ*^2^ = 27.536, *df* = 8, *P* = 0.000571; Table 1). *Microtus arvalis* and *A. flavicollis* had a significantly higher infection probability compared to *M. glareolus* (Fig. 1). There were, however, no significant effects of sex or age on individual infection probability in any of the small mammal species (Table 2).

**Table 2:** Results of a generalized linear model with binominal error distribution on individual demographic factors (sex, weight) on the probability of infection with CNM

Species	Source of variation	Coef.	SE	*z*-value	*P*-value
*M. arvalis*	Intercept	− 10.47	2.74	− 3.82	**<** **0.001**
	Sex (male)	1.81	1.07	1.68	0.092
	Weight	0.30	0.10	2.96	**0.003**
*M. glareolus*	Intercept	− 6.27	2.06	− 3.05	**0.002**
	Sex (male)	1.60	0.82	1.95	0.051
	Weight	0.16	0.07	2.32	**0.020**
*A. flavicollis*	Intercept	− 3.09	1.02	− 3.02	**0.002**
	Sex (male)	1.71	1.14	1.50	0.134
	Weight	–	–	–	–

*Notes*: Reference category for sex is female, weight was used a continuous variable. Significant factors are marked in bold; – indicates that parameter was removed during the selection process

*Abbreviation*: SE, standard error

### *Bartonella* strain characterization by sequence analysis

A representative number of 84 out of 216 (41.2%) *Bartonella*-positive samples were further processed via sequencing. A randomized algorithm was conducted to receive sequences from 35–50% of *Bartonella-*positive individuals per small mammal species, sex and country. Four *Bartonella* species, i.e. *Bartonella* sp. N40, *B. grahamii*, *B. taylorii* and *B. doshiae*, as well as uncultured *Bartonella* strains, were detected in small mammals (Table 3). Most samples yielded sequences with 98–100% similarity to uncultured *Bartonella* strains (*n* = 35) (Table 3). While *M. glareolus* were negative for uncultured *Bartonella* strain, *A. flavicollis* and *M. arvalis* yielded three different *Bartonella* uncultured strains [GenBank: MF039571 (*M. arvalis*: *n* = 24; *A. flavicollis*: *n* = 4); MF039555 (*n* = 1, *A. flavicollis* only); KU886454 (*M. arvalis*: *n* = 5; *A. flavicollis*: *n* = 1)]. In total, 21 samples showed 97–100% similarity to *B. taylorii* [GenBank: AJ269788 (*M. glareolus*: *n* = 6; *M. arvalis*: *n* = 4); AJ269784 (*S. araneus*: *n* = 5; *S. coronatus*: *n* = 4; *A. flavicollis*: *n* = 2)], 11 samples showed 100% identity with *B. grahamii* (GenBank: CP001562), and ten with a similarity of 99–100% to *B. doshiae* [GenBank: AJ269786 (*n* = 9); AF442954 (*n* = 1), all *M. arvalis*]. Seven samples showed 99–100% similarity to *Bartonella* sp. N40 [GenBank: AJ269787 (*A. flavicollis*: *n* = 2; *A. agrarius*: *n* = 2; *M. glareolus*: *n* = 1; *M. arvalis*: *n* = 1); AJ269791 (*n* = 1, *M. arvalis* only)] (Table 3). The highest diversity of *Bartonella* species was detected in *M. arvalis*, followed by *A. flavicollis* and *M. glareolus.* The diversity of *Bartonella* strains was higher in rodents (at least 4 *Bartonella* species per host species) than in shrews (only *B. taylorii*). Interestingly, *B. grahamii* was detected exclusively in *M. arvalis* originating in Germany and *B. doshiae* exclusively in *M. arvalis* from the Czech Republic.

**Table 3:** *Bartonella* species in small mammals from Germany and the Czech Republic

Small mammal family/ species^a^	No. of *Bartonella* DNA positive samples (%)	No. of *Bartonella*-positive samples selected for sequencing	No. of *Bartonella* species-positive samples				
			*B.* sp. N40	*B. grahamii*	*B. taylorii*	*B. doshiae*	*B.* uncultured
Muridae							
*Apodemus agrarius*	2 (100)	2	2	–	–	–	–
*Apodemus flavicollis*	36 (75.0)	13	2	3	2	-	6
*Apodemus sylvaticus*	4 (66.7)	3	–	3	–	–	–
Cricetidae^a^							
*Microtus arvalis*	115 (74.7)	46	2	1^b^	4	10	29
*Myodes glareolus*	35 (44.9)	11	1	4	6	–	–
Soricidae^a^							
*Sorex araneus*	19 (63.3)	5	–	–	5	–	–
*Sorex coronatus*	5 (71.4)	4	–	–	4	–	–
Total	216 (66.1)	84	7	11	21	10	35

^a^*Microtus agrestis* (*n* = 1) and *Sorex minutus* (*n* = 1) were negative for *Bartonella* spp. DNA (see Table 1) and therefore not included in this table

^b^Detected only in 1 out of 1 individual from Germany

### PCR results for *A. phagocytophilum*, CNM, *C. burnetii*, and *Babesia* spp. in small mammals

In total, 27 out of 321 (8.4%; 95% CI: 5.8–12.0%) small mammals were positive for CNM (Table 1). Samples sizes only permitted a GLM analysis for three small mammal species. Figure 1 shows that *M. glareolus* had a significantly higher probability for CNM infection compared to *M. arvalis*, but not compared to *A. flavicollis*. The two species belonging to the family Cricetidae showed an effect of weight on infection probability (Table 2). Heavier (=older) individuals were significantly more likely to be infected with CNM. Although not formally significant, sex remained in the final model and there was a trend that males were more likely to be infected compared to females. For *A. flavicollis*, only the category “sex” remained in the final model. In total, 3 out of 321 (0.9%; 95% CI: 0.2–2.8%) small mammals tested positive for *A. phagocytophilum* (*S. coronatus*, *n* = 2; *M. glareolus*, *n* = 1) (Table 1). All investigated small mammals were negative for *Babesia* spp. and *Coxiella* spp. DNA (0%; 95% CI: 0–1.4%). Regarding co-infections, double infections of *Bartonella* spp. and CNM were most frequently detected (*n* = 18; 7× in *M. glareolus*, 6× in *A. flavicollis*, 4× in *M. arvalis*, 1× in *A. sylvaticus*). Co-infections with *A. phagocytophilum* and *Bartonella* spp. occurred less often (*n* = 2; 1× in *M. glareolus*, 1× in *S. araneus*).

## Discussion

This study presents the examination of arthropod-borne pathogens such as *Bartonella* spp., *A. phagocytophilum*, CNM, *Babesia* spp. and *C. burnetii* in different small mammal species from the Czech Republic and Germany. The study was focussed on small mammals from Germany and on *Microtus* spp. from Czech Republic, which mainly inhabit pastured areas and have been neglected so far regarding their reservoir competence for arthropod-borne bacterial pathogens in central Europe. Bartonellae are zoonotic pathogens currently arranged in different phylogenetic clades with respect to their main reservoir host species. The rodent-associated bartonellae clade is by far the most diverse regarding host and *Bartonella* species [32]. The prevalence (8.1%) as well as the species variety of bartonellae in black rats (*Rattus rattus*) as well as in Norway rats (*Rattus norvegicus*) (only either *B. tribocorum* or *B. coopersplainsensis*, respectively) is observed to be low to moderate in Europe [33]. In previous European studies, *Bartonella* spp. were reported with high prevalences (16–70.6%) in *Apodemus* and *Myodes* from Sweden, Germany and Poland [6, 34, 35]. The prevalences for both rodent genera fall in line with findings from the present study. The prevalence in *M. glareolus* is expected to be lower because bank voles are known to have an immune-mediated clearance of the infection within a few months [35]. This is why it is not surprising that the prevalence in *M. glareolus* was significantly lower than in *Apodemus* and *Microtus* in the present study. Thus far, prevalences in *Microtus* voles from Poland and Austria have ranged between 14–18%; however, only a low number of individuals were tested [7, 9]. In the present study, a very high prevalence (74.7%) was detected in *Microtus* spp. which is line with recent studies from Poland and Spain (47–66.8%) [36, 37]. Individuals belonging to the genus *Microtus* were thus far not examined for immunity or the ability to resolve *Bartonella* infections. However, regarding the prevalence from the present study it seems highly unlikely that they have the ability to resolve an infection with *Bartonella* or the duration of resolving the infections seems rather long. The *Bartonella* species found in this study were likewise present in small mammals from a former study on small mammals [6]. Most *Bartonella*-positive samples yielded similarity to uncultured *Bartonella* spp. with unknown pathogenicity. This observation is in line with previous findings in other small mammals from Germany [6]. In our study, the species variety of *Bartonella* spp. was higher in rodents than in shrews. However, *B. taylorii* was found in all examined small mammal genera. This *Bartonella* species is known to be strongly associated with rodent hosts and fleas adapted to rodents such as *Ctenophthalmus nobilis* [5]. Closely related *B. taylorii*-associated strains which form in a cluster were found earlier in *Sorex* shrews from Sweden [34]. Additionally, a moderate prevalence (14.5%) for these *B. taylorii*-associated strains was detected in *S. araneus* from the UK [38]. Our study supports this hypothesis of host-specificity of *B. taylorii*-strains adapted to *Sorex* spp. as the collected specimens were solely positive for *B. taylorii*. *Bartonella grahamii* is the only *Bartonella* species of proven human-pathogenicity [3] found in rodents from the present study. Although only a small number of *Microtus* spp. originated in Germany, *B. doshiae* could exclusively be detected in these individuals, hinting that *B. doshiae* may have a rather focal distribution pattern in comparison to all other *Bartonella* species which were detected likewise in voles of both examined countries. Sex and age could not be confirmed as significant demographic factors determining individual infection status with *Bartonella* sp., which is in contrast to previous studies [35, 39].

CNM was exclusively detected in rodents, and in none of the insectivores here or in previous studies. Earlier studies showed moderate to high prevalences in *M. glareolus* and *A. flavicollis* from the Netherlands, Germany, France and Slovakia (1.8–52.7%) [14, 16, 40, 41]. Individuals belonging to the genus *Microtus* were also previously analysed for the presence of CNM in Germany, Russia, Slovakia and Sweden [10, 13, 15, 42]. However, sample sizes ranged from only two up to 24 individuals per study with a prevalence range of 0–100%. The present study shows a moderate prevalence of 4.6% in *Microtus* spp. with a more representative number of individuals (*n* = 149). Individuals belonging to the family Soricidae are assumed not to maintain CNM in the natural life-cycle [15]. As none of the examined *Sorex* spp. in our study was positive, this suggestion may be confirmed. Previous studies have reported approximately equally high prevalences of CNM in both *A. flavicollis* and *M. glareolus* [14, 16]. Moreover, our study showed that males tended to be more often infected with CNM than females. This sex-biased result has already been previously observed in *M. glareolus* and *A. flavicollis* and was explained by a higher chance of encountering CNM through a higher stress level in males as well as their higher activity radius and fights due to territorial behaviour [13]. However, another study from Slovakia could not confirm this observation [42]. Moreover, there are reports of male rodents having also higher *I. ricinus* burdens than females, which was explained by higher levels of testosterone reducing the resistance to tick infestation [43].

Interestingly, only *Sorex* spp. (5.3%) and *M. glareolus* (1.3%) were positive for *A. phagocytophilum* in the present study. High prevalences in *Sorex* spp. and *M. glareolus* have previously been reported in studies from Romania, the UK and Switzerland (9.09–19.2%) [2, 44, 45]. In particular, Bown et al. [45] emphasized the importance of *S. araneus* for the maintenance of certain *A. phagocytophilum* strains in nature. In this regard, future studies should focus on a more thorough investigation of *Sorex* spp. as potential reservoirs, as our study also found high prevalences in *Sorex* spp. In contrast, all other captured small mammal species from the present study presumably only play a minor or no role in the maintenance of *A. phagocytophilum* in its natural life-cycle in central Europe.

In the present study, neither *Babesia* nor *C. burnetii* were found in the small mammals leading to the conclusion that the captured small mammal species may play only a subordinate role in their transmission life-cycle. Pluta et al. [18] also reported a lack of *C. burnetii* in small mammals from endemic areas in southern Germany. Nonetheless, DNA of *C. burnetii* was detected by low prevalence rates in brown and in black rats at livestock farms from the Netherlands [46]. In Spain, *C. burnetii* was further found in a few small mammals collected from a sheep farm with reported Q fever outbreaks [47]. However, these rodents may have acquired the infection *via* indirect contact with infected sheep rather than through tick bites. In earlier studies, *Babesia* was found in *Microtus* and other small mammal species with a low to moderately high prevalence in Switzerland, Germany and Poland (0.4–14.17%) [48–50]. Still, a lack of *Babesia* spp. has also been reported in *A. flavicollis* and *M. glareolus* from Poland [51] which is in line with our findings. Moreover, the overall prevalence for *B. microti* in *Ixodes ricinus* ticks from central and eastern Europe is also known to be rather low (0.5–13%) [52].

## Conclusions

To our knowledge, this study shows for the first time a very high prevalence of *Bartonella* in *M. arvalis* from the Czech Republic. The prevalence for flea-borne bartonellae was higher than for tick-borne pathogens in *M. arvalis* in contrast to other tested common rodent species such as *M. glareolus*. The reason may be that *Microtus* spp. are more likely to live in grassland and agricultural areas than in urban or sylvatic regions where ticks are more prevalent. The species diversity of *Bartonella* spp. was higher in rodents than in shrews. *Sorex* spp. seem to be only relevant for the maintenance of non-pathogenic *B. taylorii*. Interestingly, only *Sorex* spp. and *M. glareolus* were positive for *A. phagocytophilum* in the present study, suggesting their potential importance for the maintenance of certain *A. phagocytophilum* strains in nature.

## Additional file

**Additional file 1: Table S1.** Details on primers and PCR assays used for the detection of vector-borne pathogens in rodents from Germany and the Czech Republic. *Abbreviations*: C, conventional PCR; RT, real-time PCR; ITS, intergenic spacer.

## Data Availability

The data supporting the conclusions of this article are included within the article and its additional file. The raw data used and/or analysed during the present study are available from the corresponding author upon reasonable request.

## Abbreviations

BLASTn: Basic Local Alignment Search Tool nucleotide
CI: confidence interval
CNM: “*Candidatus* Neoehrlichia mikurensis”
CVBD: Canine vector-borne diseases
df: degrees of freedom
GLM: Generalized linear model
icd: Isocitrate dehydrogenase
PCR: polymerase chain reaction
