# Supplementary material for: Highly prevalent bartonellae and other vector-borne pathogens in small mammal species from the Czech Republic and Germany
Source: Parasit Vectors. 2019 Jul 3;12:332. doi: 10.1186/s13071-019-3576-7 (PMC6610854; doi:10.1186/s13071-019-3576-7)
Supplement: Supplementary file 1 — Additional file 1: Table S1. Details on primers and PCR assays used for the detection of vector-borne pathogens in rodents from Germany and the Czech Republic. Abbreviations: C, conventional PCR; RT, real-time PCR; ITS, intergenic spacer. [file 13071_2019_3576_MOESM1_ESM.docx]

**Additional file 1: Table S1.** Details on primers and PCR assays used for the detection of vector-borne pathogens in rodents from Germany and the Czech Republic.

| **Pathogen** | **PCR  type** | **Primer name** | **Primer/probe sequences 5'-3'** | **Gene (amplicon size in bp)** | **Reference** |
| --- | --- | --- | --- | --- | --- |
| *Anaplasma phagocytophilum* | RT | ApMsp2f | ATGGAAGGTAGTGTTGGTTATGGTATT | *msp2*  (77) | [25, 29] |
|  |  | ApMsp2r | TTGGTCTTGAAGCGCTCGTA |  |  |
|  |  | ApMsp2p | FAM-TGGTGCCAGGGTTGA GCTTGAGATTG-BHQ1 |  |  |
| *Babesia* spp. | C | BJ1 | GTCTTGTAATTGGAATGATGG | *18S rRNA*  (411–452) | [27] |
|  |  | BN2 | TAGTTTATGGTTAGGACTACG |  |  |
| *Bartonella* spp. | C | Ba325s | CTTCAGATGATGATCCCA AGCCTTCTGGCG | *16S-23S rRNA* (ITS)  (453–780) | [25, 26] |
|  |  | Ba1100as | GAACCGACGACCCCCTGCTTGCAAAGC |  |  |
| *“Candidatus* Neoehrlichia mikurensis” | RT | NMikGroEL F2 | CCTTGAAAATATAGCAAGATCAGGTAG | *groEL* (99) | [14] |
|  |  | NMikGroEL rev1 | CCACCACGTAACTTATTTAGCACTAAAG |  |  |
|  |  | NMikGroEL rev2 | CCACCACGTAACTTATTTAGTACTAAAG |  |  |
|  |  | NMikGroEL-P2a | FAM-CCTCTACTAATTATTGCT GAAGATGTAGAAGGTGAAGC-BHQ1 |  |  |
| *Coxiella burnetti* | RT | 439F | CGTTATTTTACGGGTGTGCCA | *icd*  (76) | [52] |
|  |  | 514R | CAGAATTTTCGCGGAAAATCA |  |  |
|  |  | 464TM | FAM-CATATTCACCTTTTCAGGCGTTTTGACCGT-TAMRA-T |  |  |

*Abbreviations*: C, conventional PCR; RT, real-time PCR; ITS, intergenic spacer
